# Supplementary material for: Geometric reduction of dynamical nonlocality in nanoscale quantum circuits
Source: Sci Rep. 2016 Jan 6;6:18827. doi: 10.1038/srep18827 (PMC4702143; doi:10.1038/srep18827)
Supplement: Supplementary Information [file srep18827-s1.pdf]

Supplementary Information: Geometric reduction of dynamical nonlocality in nanoscale quantum circuits

E. Strambini†, K.S. Makarenko†, G. Abulizi, M.P. de Jong and W.G. van der Wiel*

NanoElectronics Group, MESA+ Institute for Nanotechnology, University of Twente, PO Box 217, 7500 AE, Enschede, The Netherlands

† These authors contributed equally to this work.
* e-mail: W.G.vanderWiel@utwente.nl

1. Characterization of Aharonov-Bohm Interferometers

1.1. Evaluation of the Coherence Length

Figure S1 shows the fast Fourier transform (FFT) of the magneto-resistance oscillations measured through the device in Fig. 1a in the local and the nonlocal configurations, after subtracting a polynomial fit (to remove the aperiodic background fluctuations, UCF). Six clear peaks corresponding to the h/ne (where n = 1,2,…6 is the harmonic index) harmonics can be observed.

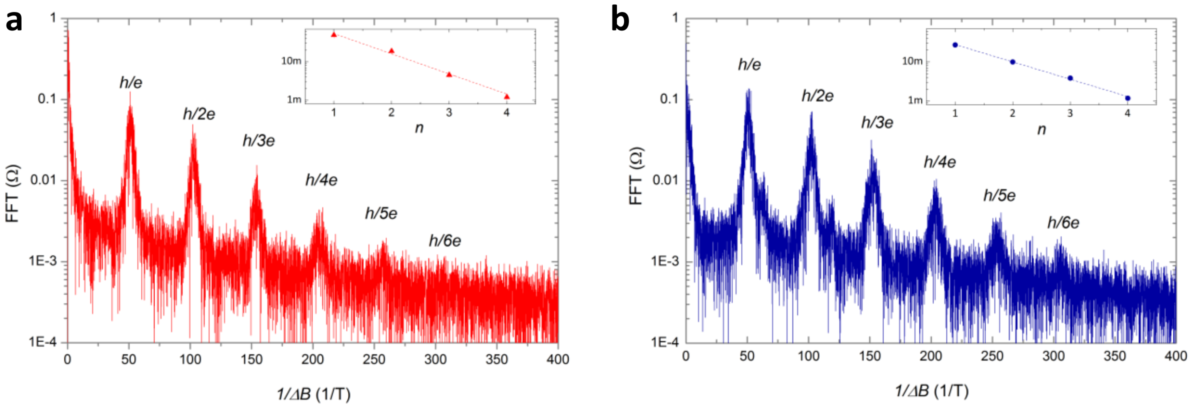

Figure S1 | Spectral analysis of the AB oscillations. a-b, Representative FFT of R_L (**a**) and R_{NL} (**b**) of the interferometer in Fig. 1a, showing up to six harmonics. Insets show the amplitude of the first four peaks of the magneto-resistance, represented in **a** and **b**, versus the harmonic index (n) (dots) and the exponential fit (lines) used to extract the coherence length. (temperature 40 mK, 30 nA ac excitation current).

The amplitude of the AB oscillations decays exponentially with the coherence length of the system according to these relations [S1]:

$$\text{for } R_L: \qquad A \propto e^{-0.6\frac{nC}{l_\varphi}}, \qquad \text{(S1)}$$

for R_{NL}: $$A \propto e^{-\left(0.6\frac{nC}{l_\varphi}+1.1\frac{d}{l_\varphi}\right)}, \quad (S2)$$

where C is the circumference of AB interferometer, d is the total length of the side arms, l_φ is coherence length. Based on Eqs. (S1)-(S2) we can extract the ratio between C and l_φ by fitting the peak amplitude in the Fourier transform (see insets of Fig. S1a and b). From the periodicity of the AB oscillation we extract the area of the ring used to estimate the average circumference C from the hexagonal geometry of the ring. After this calculation we obtained a coherence length l_φ of ~1.5 μm for both local and nonlocal configurations.

1.2. Temperature Dependence of the local and nonlocal interference

From the data analysis reported in the previous section we estimated the temperature dependence of the coherence length reported in Fig. S2. In agreement with previous characterization of the coherence length in Au samples [S2], below 1K the coherence length is not affected by the temperature of the system.

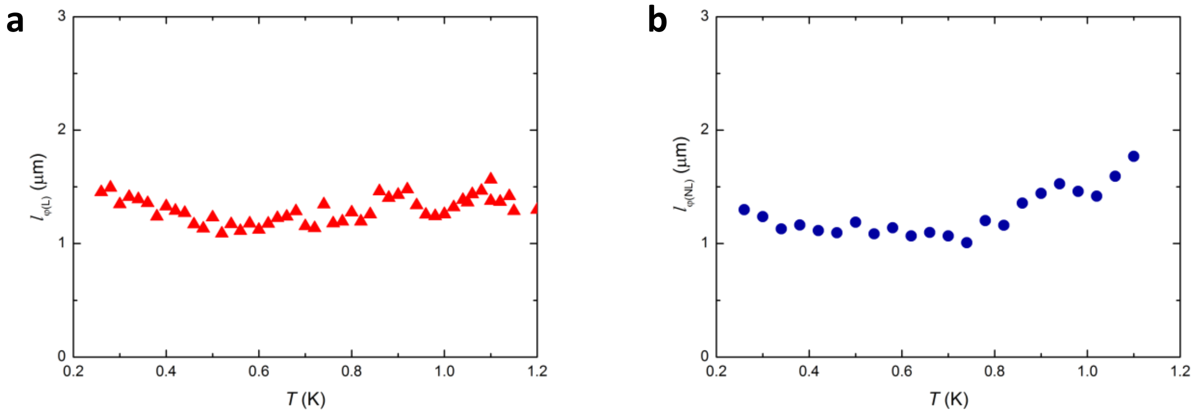

Figure S2| Temperature dependence of the coherence length of the AB interferometer in Fig.1: for the local (**a**) and nonlocal (**b**) geometries (30 nA ac excitation current).

By contrast, despite the constant coherence length, in the same temperature range the amplitude of the oscillations is decreasing, as demonstrated in Fig. S3 showing the temperature dependence of the first four FFT peaks. We than ascribe this damping to phase averaging due to thermal broadening [S3, S4].

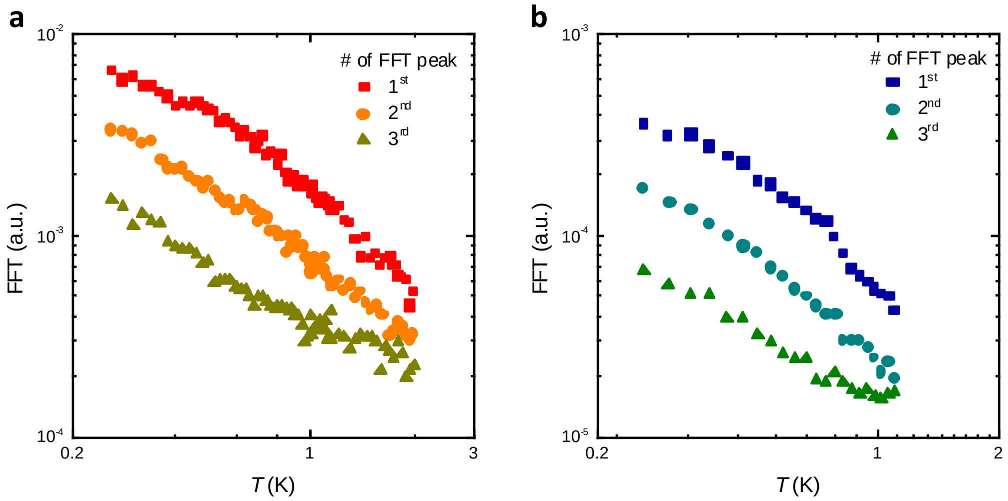

Figure S3| Temperature dependence of the AB oscillation amplitude: dependence of the *h/ne* oscillations for the local (**a**) and nonlocal (**b**) geometries (30 nA ac excitation current).

1.3. Excitation Current Dependence of the AB Oscillation Amplitudes

Figure S4 shows an excitation current (I_{ac}) dependence of the AB oscillation amplitudes for the 1st, 2nd and 3rd FFT peaks, measured in the local configuration. The amplitude of the FFT peaks does not change for excitation currents below ~50 nA (see the crossing point of two red dashed guiding lines in Fig.S4). From this I_{ac} value and the AB ring's resistance (~282 Ω) the corresponding energy broadening is ΔE~14 μeV from which is possible to estimate an effective electron temperature T_e≃50 mK (ΔE=3.5 kT_e). Above this point the peak amplitudes decay due to phase averaging or heating induced by the excitation current.

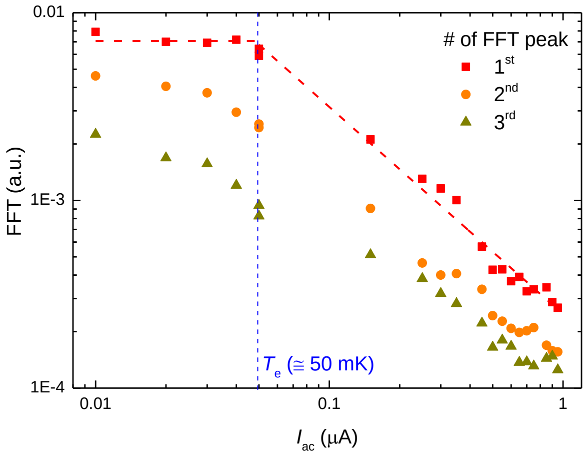

Figure S4| Excitation current dependence of the 1st, 2nd and the 3rd FFT peaks of the AB oscillations measured for the AB ring in Fig. 1a in the local configuration (T=40 mK).

1.4. Dependence of the AB oscillations on the Ring Size

The AB period is determined by the ring's enclosed area S_{in}, $\Delta B = h/eS_{\text{in}}$ [S5]. Figure S6 shows electrical measurements on two AB interferometers of different size (490 nm and 590 nm in diameter) for local (Fig. S6a) and nonlocal (Fig.S6b) configurations. Calculated from the geometry of the AB rings, the oscillation frequencies $1/\Delta B = (e/h)S_{\text{in}}$ are (52 ± 2) and (75 ± 2) T^{-1} for the (490 ± 10) and (590 ± 10) nm diameter interferometers, respectively. These values are in agreement with the periodicities extracted from the FFT peaks (~51 and ~74 T^{-1} for 490 and 590 nm, respectively) (Fig. S6). The coherence length, estimated from the damping of the high-order harmonics (see section 1.1), is ~1.5 μm for both rings and measurement configurations.

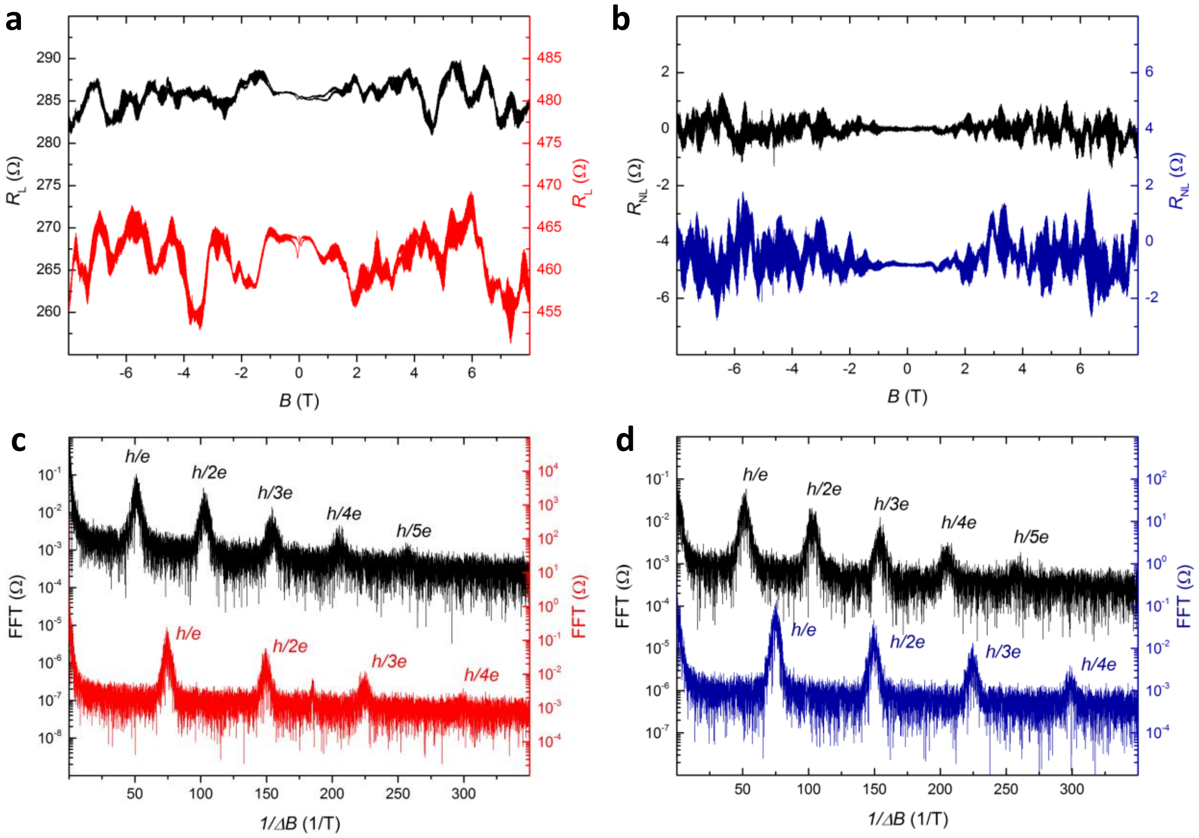

Figure S5| Comparison between AB interferometers of two different size for local and nonlocal setups. a, **b**, Magneto-resistance of two Au AB interferometers with a diameter of ~490 nm (black curves) and ~590 nm (red and blue curves) measured in local (**a**) and nonlocal (**b**) geometries. **c**, **d**, Fourier transform of the data presented in **a** and **b**, respectively.

1.5. *Local and Nonlocal Measurements in the Multi-Terminal Quantum Interferometer*

Figure S6 shows electrical measurements through the multi-terminal AB interferometer shown in Fig.2a. The local resistance (R_L) does not depend on the total length of side arms *d* (Fig.S6c) while the nonlocal resistance (R_{NL}) is strongly dependent on the total length of side arms (Fig.S6c).

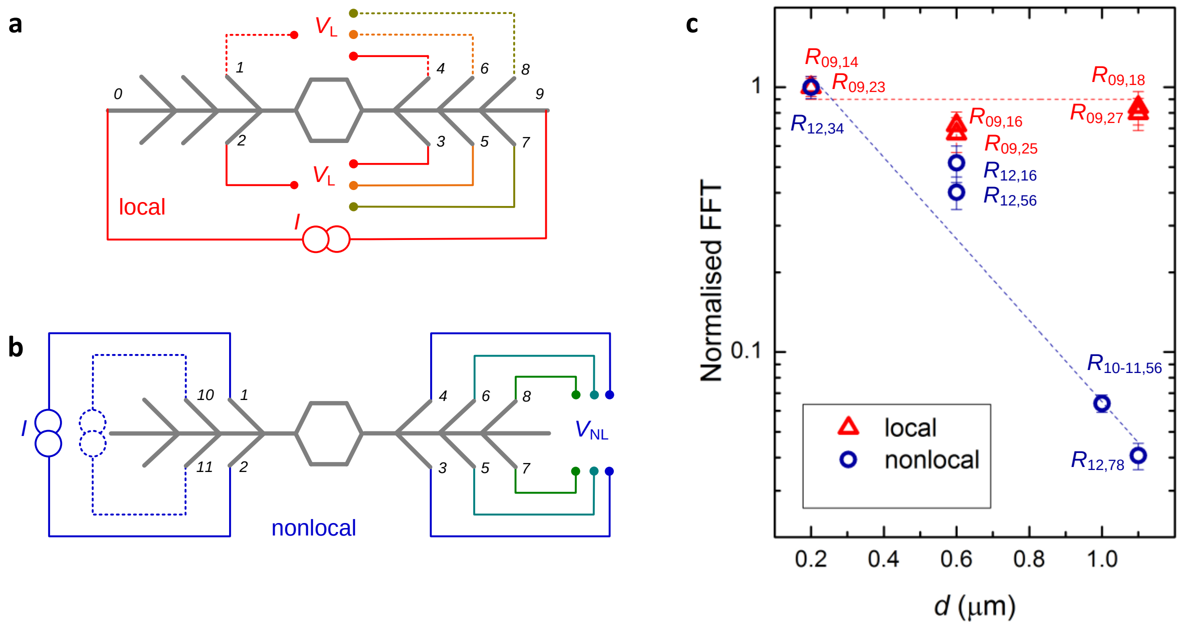

Figure S6| Dependence of R_L and R_{NL} on the total length of the side arm. a-b, Schematics of the multi-terminal Au AB interferometer for local (**a**) and nonlocal (**b**) configurations. **c**, Normalised Fourier transform of the measured R_L (red) and R_{NL} (blue): the amplitude of the *h/e* oscillations is decreasing with increasing of the total length of the side arms (*d*) for the nonlocal configuration while for the local configuration the amplitude almost does not change.

2. THEORETICAL MODEL

2.1. *Scattering Matrix Model of the Nonlocal AB Effect*

Here, we describe in detail the theoretical model used to simulate the local and nonlocal resistance of a four-terminal AB ring. Following the Landauer-Büttiker (LB) formalism [S6], the transport properties of a 4-terminal device can be fully described by a unitary 4×4 scattering matrix (t_{ij}) representing the amplitude probabilities for an electron injected in the terminal *i* to be transmitted to terminal *j*. The four-wire resistances of the device are then

extracted from the coefficients of the probability matrix $T_{ij} = |t_{ij}|^2$ according to the LB analytical formula [S6]:

$$R_{nm,kl} = (h/2e^2) \cdot (T_{km}T_{ln} - T_{kn}T_{lm})/D \qquad \text{(S3)}$$

where h is the Plank constant, e is the electronic charge, and D is a quantity including all of the T_{ij}'s coefficients.

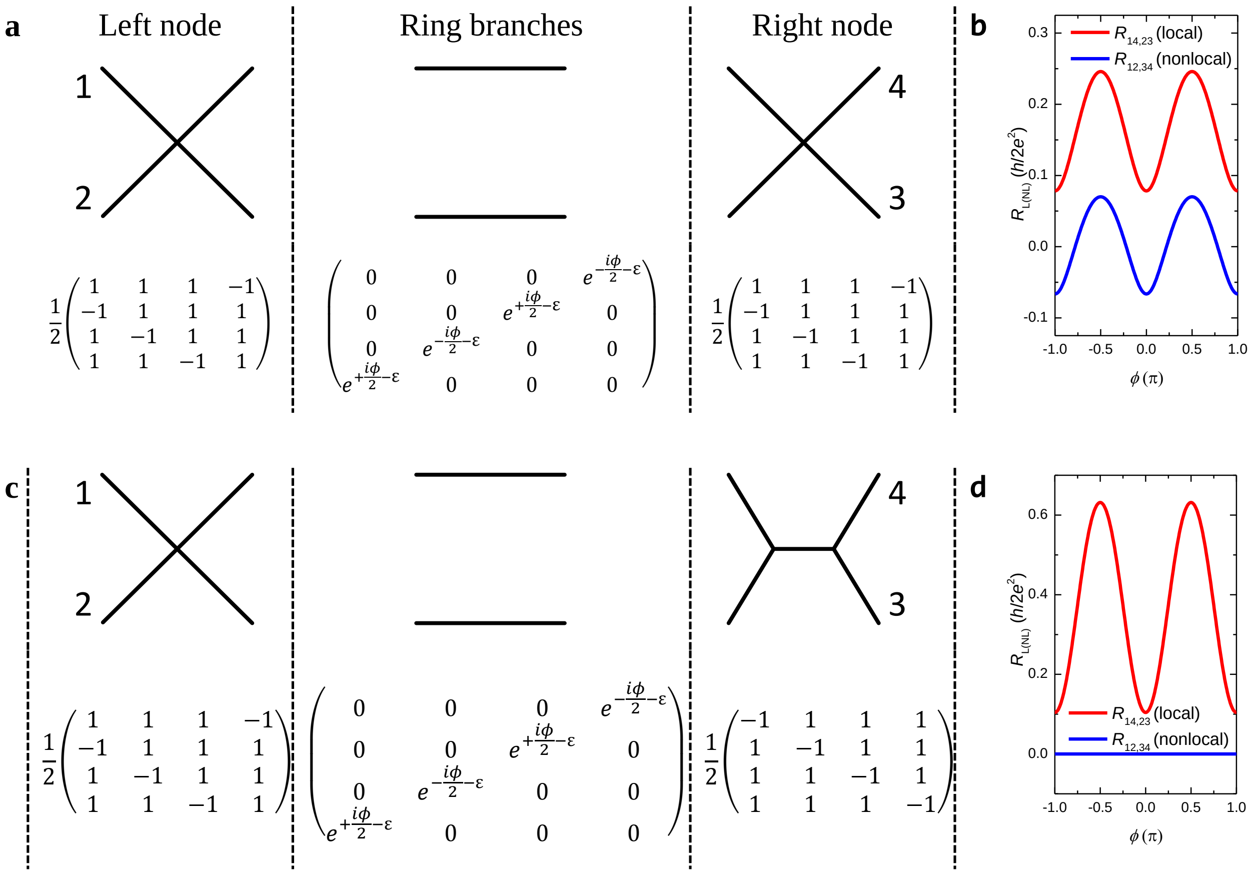

Figure S7| Representation of the 4-terminal AB ring in the LB formalism without and with a side arm, panel **a** and **b**, respectively. Local and nonlocal resistances simulated for the two different models as a function of the AB phase shift ϕ and for the decoherence damping of ε=0.5.

The overall scattering amplitudes s_{ij} of this system can be calculated from the scattering matrices of the individual building blocks representing the 4-terminal ring, i.e. two 4×4 scattering nodes and the two branches of the ring, according to the scheme presented in Fig. S7a. The scattering nodes can be represented by a unitary 4×4 real matrix, while we include all the complex phase shifts in the scattering matrices describing the two branches of the ring. To better represent a metallic node, we use a fully symmetric scattering centre

(shown in Fig. S7a). Different asymmetries in the scattering amplitudes have been investigated, all of them giving similar results.

The scattering matrix of the two branches of the ring is composed of a simple phase shifter of half of the AB phase (we only consider the phase shift due to the magnetic vector potential), $\pm e^{\frac{i\phi}{2}}$ per branch (symmetric ring), and a small damping ($e^{-\varepsilon}$) to simulate also the loss of coherence usually preventing higher order interference.

With this choice of scattering matrices, we simulate the local and nonlocal resistances as a function of the magnetic phase ϕ. Figure S1b shows how this simple model can catch all the essential physics of the local and nonlocal interference observed in our experiment. Both signals show characteristic AB oscillations. The AB oscillations are superimposed on top of an offset in case of the local configuration, while with almost zero average in the nonlocal case.

However, the model gives a drastically different outcome if we replace one (or both) of the two scattering nodes with a side arm to approximate the actual geometry of our experimental device. When using this approximation, with the corresponding scattering matrices given in Fig. S7c all the nonlocal resistances become zero, while the local signal remains unchanged (Fig. S7d).

This quenching of the nonlocal signal induced by the reduction of channels in a side arm is not dependent on the specific choice of the scattering nodes, but is a generic property of any quantum network incorporating a transport channel reduction. In the following we will demonstrate this property for a generic quantum transport network.

2.2. *Demonstration of the Quenching of the Nonlocal Signal for a Generic Network*

A generic quantum network composed of a one-dimensional constriction is represented in the LB formalism by the superposition of two generic scattering matrices ($t_{ij}^{Left} \in U\,(N+1)$ and $t_{ij}^{Right} \in U\,(M+1)$) connected by a single arm, as schematically shown in Fig S8. The composition of these two scattering matrices results in a singularity of the elements of the total scattering matrix $t_{ij} \in U\,(N+M)$. Assuming for clarity that index 1 corresponds to the reduced common path for both the matrices, the transmission amplitude of the total scattering have the simple analytical form [S7]:

$$t_{ij} = t_{1(i-N)}^{Right} t_{j1}^{Left} / (1 - t_{11}^{Right} t_{11}^{Left}) \text{ for } i > N \text{ and } j < N, \qquad \text{(S4)}$$

$$t_{ij} = t_{(j-N)1}^{Right} t_{1i}^{Left} / (1 - t_{11}^{Right} t_{11}^{Left}) \text{ for } i < N \text{ and } j > N; \quad \text{(S5)}$$

These equations, linear in the transmission amplitude of the two scattering matrices, allow for the following commutation rule: $t_{ij}t_{kl} = t_{il}t_{kj}$. As a consequence of this rule all the nonlocal resistances measured across the constriction are singular $R_{nmkl} =\propto T_{km}T_{ln} - T_{kn}T_{lm} = |t_{km}t_{ln}|^2 - |t_{kn}t_{lm}|^2 = 0$; $\forall\, n,m < N$ and $k,l > N$ (or $\forall\, n,m > N$ and $k,l < N$).

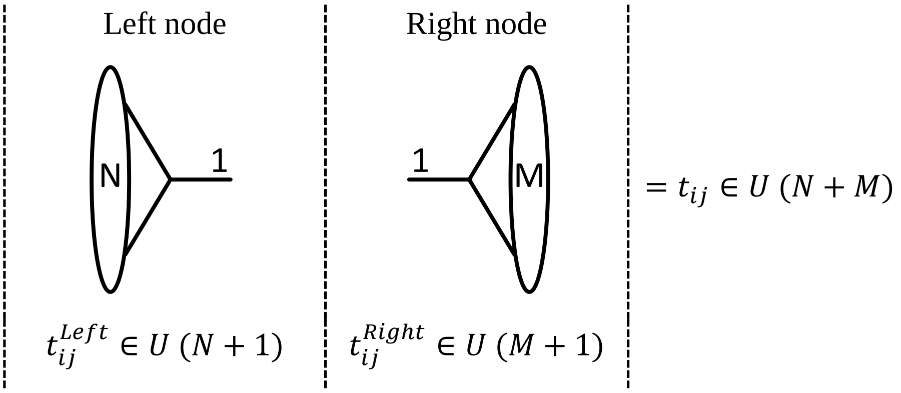

Figure S8| Schematic showing a generic quantum network composed of a one-dimensional constriction is represented in the LB formalism by the superposition of two generic scattering matrices ($t_{ij}^{Left} \in U\ (N+1)$ and $t_{ij}^{Right} \in U\ (M+1)$) connected by a single arm.

REFERENCES

[S1] D. P. DiVincenzo and C. L. Kane, "Voltage fluctuations in mesoscopic metal rings and wires," *Physical Review B,* vol. 38, pp. 3006-3015, 08/15/ 1988.

[S2] P. Mohanty, E. M. Q. Jariwala, and R. A. Webb, "Intrinsic Decoherence in Mesoscopic Systems," *Physical Review Letters,* vol. 78, pp. 3366-3369, 04/28/ 1997.

[S3] A. D. Stone, "Magnetoresistance Fluctuations in Mesoscopic Wires and Rings," *Physical Review Letters,* vol. 54, pp. 2692-2695, 06/24/ 1985.

[S4] P. A. Lee and A. D. Stone, "Universal Conductance Fluctuations in Metals," *Physical Review Letters,* vol. 55, pp. 1622-1625, 10/07/ 1985.

[S5] Y. Aharonov and D. Bohm, "Significance of Electromagnetic Potentials in the Quantum Theory," *Physical Review,* vol. 115, pp. 485-491, 08/01/ 1959.

[S6] M. Büttiker, "Four-Terminal Phase-Coherent Conductance," *Physical Review Letters,* vol. 57, pp. 1761-1764, 10/06/ 1986.

[S7] S. Datta, *Electronic transport in mesoscopic systems*: Cambridge university press, 1997.
